# Patterns of relapse in diffuse intrinsic pontine glioma after convection-enhanced delivery of ^124^I-omburtamab

**DOI:** 10.1093/noajnl/vdaf128

**Published:** 2025-07-19

**Authors:** Evan D Bander, Andrew L A Garton, Luca Pasquini, Anne S Reiner, Onur Yildirim, Ahmet T Ilica, Maria Donzelli, Sofia Haque, Mark M Souweidane

**Affiliations:** Department of Neurological Surgery, Memorial Sloan Kettering Cancer Center, New York City, New York, USA; Department of Neurological Surgery, Memorial Sloan Kettering Cancer Center, New York City, New York, USA; Radiology, Memorial Sloan Kettering Cancer Center, New York City, New York, USA; Epidemiology and Biostatistics, Memorial Sloan Kettering Cancer Center, New York City, New York, USA; Radiology, Memorial Sloan Kettering Cancer Center, New York City, New York, USA; Department of Neurological Surgery, Memorial Sloan Kettering Cancer Center, New York City, New York, USA; Department of Neurological Surgery, Memorial Sloan Kettering Cancer Center, New York City, New York, USA; Radiology, Memorial Sloan Kettering Cancer Center, New York City, New York, USA; Department of Neurological Surgery, Memorial Sloan Kettering Cancer Center, New York City, New York, USA

**Keywords:** convection-enhanced delivery, DIPG/DMG, phase I, recurrence, theranostic

## Abstract

**Background:**

Diffuse intrinsic pontine glioma (DIPG) carries a high mortality rate and lacks effective treatment options with a median overall survival (OS) of 8–12 months. Convection-enhanced delivery (CED) has demonstrated safety in phase I trials, but efficacy is indeterminate. Evaluating anatomic patterns of relapse may aid in determining therapeutic efficacy of local CED drug delivery strategies.

**Methods:**

Sixty-three children with DIPG were retrospectively reviewed for first radiographic progression. All patients were treated using conventional external beam radiation (EBRT) and 31 were treated with CED of radiolabeled 124-iodine-omburtamab (NCT01502917). Anatomic patterns of initial progression were coded by independent neuroradiologists. OS and cumulative incidence of progression at each anatomic site were assessed in a competing risk analysis with death as a competing variable and were stratified based on CED treatment.

**Results:**

Median OS was 14.67 months for the cohort. Patients receiving CED demonstrated higher rates of progression in general, when considering progression at all anatomical sites (HR 1.79, *P* = .047); no significant difference was found in OS when stratified by CED treatment (*P* = .22). However, CED treatment was associated with significantly lower cumulative incidence of local pontine and medullary progression (HR: 0.42, *P* = .03; HR 0.14, *P* = .01, respectively) relative to non-CED-treated patients.

**Conclusions:**

Anatomically defined patterns of relapse provide evidence for locoregional control in children with DIPG treated with radioimmunotherapy administered by CED. Future CED or local surgical therapy trials can benefit from including detailed patterns of relapse as a prospective outcome.

**Key Points**

- Sixty-three children with diffuse intrinsic pontine glioma (DIPG) were evaluated for radiographic progression of disease, stratified by treatment with convection-enhanced delivery (CED).
- Patients receiving CED demonstrating lower cumulative incidence of local pontine and medullary progression relative to non-CED-treated patients.

**Importance of the Study**

The results of this study are some of the first to provide in-human use of a theranostic ^124^I radiopharmaceutical and query the efficacy of convection-enhanced delivery (CED) for establishing locoregional control as compared to distant recurrences. Studying patterns of relapse in patients undergoing CED for the treatment of CNS malignancy will prove to be an important prospective outcome in the treatment of CNS malignancies.

Median survival for patients with diffuse midline glioma (DMG)/diffuse intrinsic pontine glioma (DIPG) is estimated at 8–12 months, with fewer than 25% of patients surviving 1 year after diagnosis.^1–3^ Median time to progression after external beam radiotherapy (EBRT) is 5–6 months.^4–6^ Systemic delivery of therapies has had minimal benefit in clinical trials due to limitations of systemic toxicity.^7,8^ Convection-enhanced delivery (CED) into the brainstem, which can bypass the blood–brain barrier (BBB) may hold promise as an attempt to achieve local control; however, data regarding long-term outcomes are still limited.^9–15^ Determining efficacy of locally delivered agents is confounded by separating efficacy at the targeted/treatment site with progression and recurrence beyond the treated site. Assessing overall survival (OS) may not be sensitive enough to detect local efficacy in clinical trials. Furthermore, few studies have examined specific anatomic/radiographic patterns of relapse following conventional or experimental therapy for DIPG, with the most detailed assessments limited to local (within brainstem, peduncles, or cerebellum) or distant failure.^16^ Patterns of relapse in other forms of high-grade glioma following local or systemic therapy have been previously studied (eg, local, marginal, or distant recurrence after resection/radiation and rates of local recurrence and CNS dispersion following bevacizumab^17,18^). However, local therapies for DIPG have been previously limited to external beam radiation and, therefore, CED treatment directly to the pons creates a unique opportunity for identifying specific anatomic locations of locoregional and distant relapse after a novel localized treatment. We therefore reviewed a cohort of DIPG patients at a single institution to better describe anatomic/radiographically defined patterns of relapse and to further analyze a subgroup of children treated with CED following conventional EBRT to demonstrate that CED can effect locoregional patterns of relapse.

## Methods

### Patient Selection

Patients in this study were enrolled between 2012 and 2021 in a phase I single-center clinical dose-finding trial (ClinicalTrials.gov Identifier: NCT01502917) at Memorial Sloan Kettering (MSK) using ^124^I-omburtamab monoclonal antibody (Y-mAbs therapeutics) administered by CED, as previously described.^19–22^ All subjects were radiographically diagnosed with DIPG and were previously treated with standard-of-care EBRT. Patients in dose level 5 and above (*n* = 31) were included for this retrospective analysis, given the greater likelihood of clinical activity of the treatment at these dose levels.^19^ A second cohort of children (age < 18) diagnosed between 2010 and 2018 with DIPG and followed/treated at MSK, treated with standard-of-care EBRT, with at least 2 follow-up magnetic resonance imagings (MRIs) after EBRT, but not on trial NCT01502917 were retrospectively identified (*n* = 32). Patients were excluded for imaging at diagnosis, post-EBRT, or at follow-up that was unavailable for review by neuroradiologists or if the patient did not have record of receiving standard-of-care EBRT after diagnosis. These 2 cohorts were used for the ensuing analysis. In both the clinical trial and control cohorts, DIPG was a clinical/radiographic diagnosis and neither histology/tissue nor genetic/molecular diagnostics were required for inclusion. Eleven patients (34%) in the control cohort had tissue diagnosis confirming DIPG, with only 3 reports testing and confirming presence of an H3K27M mutation. Sixteen patients (51.6%) in the CED cohort had tissue diagnosis, with 14 confirming H3K27M mutation and 2 not reporting on H3K27 status. IRB approval was obtained for retrospective analysis. Retrospective chart and radiology review were used to assess demographic data, CED infusion data, recurrence patterns, follow-up, and survival.

### Radiographic Analysis

Based on the WHO criteria from 2021, a diagnosis of pediatric-type DMG H3K27-altered requires molecular confirmation; however, at the time of study initiation, DIPG remained a clinical diagnosis, and biopsy was not standard practice as alluded to in that only 50% of our cohort had molecular diagnosis. A consensus clinical diagnosis was therefore made by a multidisciplinary pediatric neuro-oncology team based on clinical evidence and MRI. MRI was reviewed by nonblinded, certified neuroradiologists to assess disease progression over time and treatment effects. Tumor features including imaging characteristics and tumor extension at 3 different MRI time points were evaluated. For the patient cohort, we considered the following time points: diagnostic MR scan (t1), pre-CED treatment/post-EBRT (t2); first progression scan (t3—representing the first MR showing tumor progression after treatment). For the control cohort, we considered the same time points, apart from t2, which was set immediately after the conclusion of EBRT.

**Figure 1.**
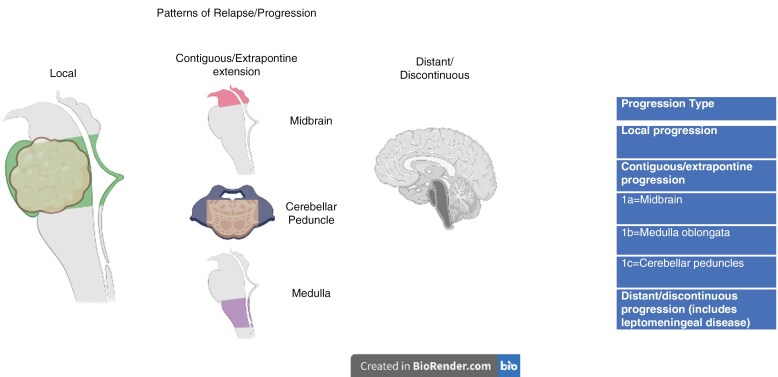
Diagrammatic illustration of patterns of anatomic/radiographic relapse.

**Figure 2.**
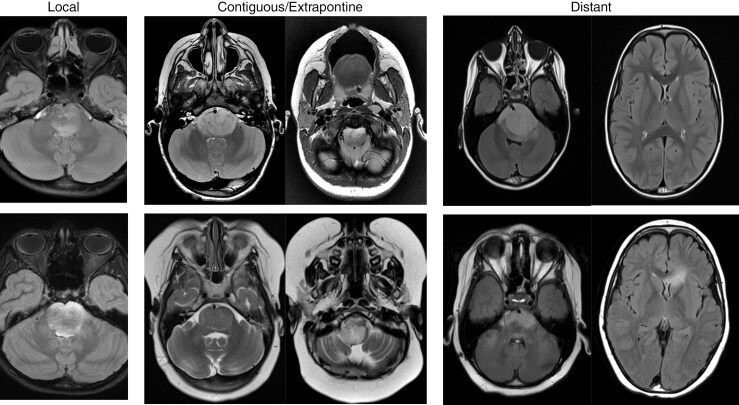
Representative axial MRI FLAIR or T2 sequences from 3 CED patients at 2 time points (t1 or t2 vs T3) demonstrating different anatomic/radiographic subtypes of progression. (Left) Local progression within the pons. (Middle) Improved local/pontine disease with new contiguous/extrapontine progression within the medulla. (Right) Improved local/pontine disease with distant progression in the corpus callosum. CED, convection-enhanced delivery.

The evaluation at t1 and t2 included: tumor location, extrapontine extension (EPE) defined as in Makepeace et al.,^23^ presence of diffusion restriction, and contrast enhancement within the tumor. The evaluation at t3 included: progression type based on RAPNO criteria,^24^ presence of diffusion restriction, and contrast enhancement within the tumor. Each MRI was analyzed using anatomic and radiographic failure criteria to denote location of progression, as follows: local, contiguous (with specification of medulla, midbrain, and/or middle cerebellar peduncles [MCPs]), and/or distant (any location beyond the brainstem and not otherwise described by the preceding criteria) (Figures 1 and 2). Local (pontine) progression of disease was defined as a >25% increase in the 2-dimensional (2D) perpendicular diameters of the tumor lesion by imaging measured on T2/FLAIR sequences.^24^ Contiguous and distant progression were defined as any new evidence of tumor disease in the respective sites with ≥25% increase in the 2D product of perpendicular diameters on T2/FLAIR sequences. The evaluation was conducted by 3 experienced neuroradiologists and the final scoring was obtained as per consensus.

### Statistical Analysis

Descriptive statistics such as proportions, means, and standard deviations were used to characterize the cohort. OS was assessed with Kaplan–Meier methodology and compared between groups using the log-rank test. Follow-up was calculated from t1 as described above until death (event) or last follow-up (censored). Cumulative incidence of progression at each anatomic site was assessed using univariable and multivariable cause-specific competing risk regression with death as a competing event and stratification by CED treatment. Multivariable models adjusted for EPE on MRI at t1 which was statistically significantly different by CED treatment group. All tests were 2-sided with a preestablished level of statistical significance < .05. All statistical analyses were performed in SAS v9.4 (The SAS Institute) and R v4.1.3 (The R Foundation for Statistical Computing).

## Results

### Demographics and Survival

Sixty-three children clinically/radiographically diagnosed and treated for DIPG at MSK between 2010 and 2022 were included in this analysis. All patients (100%) were treated using conventional EBRT and 31 (49%) were treated with CED of radiolabeled 124-iodine-omburtamab following EBRT (NCT01502917). The entire cohort mean age was 7.1 (± 3.4 years) and 60% of the patients were female (Table 1). At time of diagnosis, baseline imaging of the tumors demonstrated presence of EPE in 76% of patients, diffusion restriction was present in 33%, and contrast enhancement in 53%.

**Table 1.** Characteristics overall and by group

		Overall cohort					Treatment group					Control group					
Variable	Level	*N*	%	Mean	SD	Range	*N*	%	Mean	SD	Range	*N*	%	Mean	SD	Range	*P* value*
Age at diagnosis	Continuous, years	63	100	7.1	3.4	2.4–17.3	31	100	7.8	3.9	2.4–17.3	32	100	6.5	2.8	2.6–11.8	.22
Time from EBRT to first scan with progression	Continuous, days	50	79	133.1	109.6	6–518	27	87	114.2	94.6	25–404	23	72	155.3	123.4	6–518	.24
Sex	Male	25	40				14	45				11	34				.38
	Female	38	60				17	55				21	66				
EPE on MRI at Dx	No	15	24				11	35				4	13				.03
	Yes	48	76				20	65				28	88				
DWI on MRI at Dx	No	38	67				19	73				19	61				.35
	Yes	19	33				7	27				12	39				
	Unknown	6					5					1					
T1CE on MRI at Dx	No	28	47				15	52				13	42				.45
	Yes	32	53				14	48				18	58				
	Unknown	3					2					1					

EBRT, external beam radiotherapy; EPE, extrapontine extension; DWI, diffusion weighted imaging; T1CE, T1 contrast enhancing.

*P value does not include unknown categories.

With respect to the patients receiving CED, infusion doses ranged from a prescribed dose of 2.5 to 10 mCi (*n* = 3 at 2.5 mCi, *n* = 3 at 3.25 mCi, *n* = 15 at 4.0 mCi, *n* = 6 at 6 mCi, *n* = 3 at 8 mCi, *n* = 1 at 10 mCi). Maximum flow rates ranged from 3.5 to 10.0 μL/min. The median infusion volume among CED subjects was 4.4 mL. Twenty-six patients (83.8%) underwent a single infusion, 4 patients (12.9%) received 2 infusions (median infusion volume: 4.07 mL), and 1 patient (3.2%) received 3 infusions (volume: 4.135 mL), as previously described.^20^

**Figure 3.**
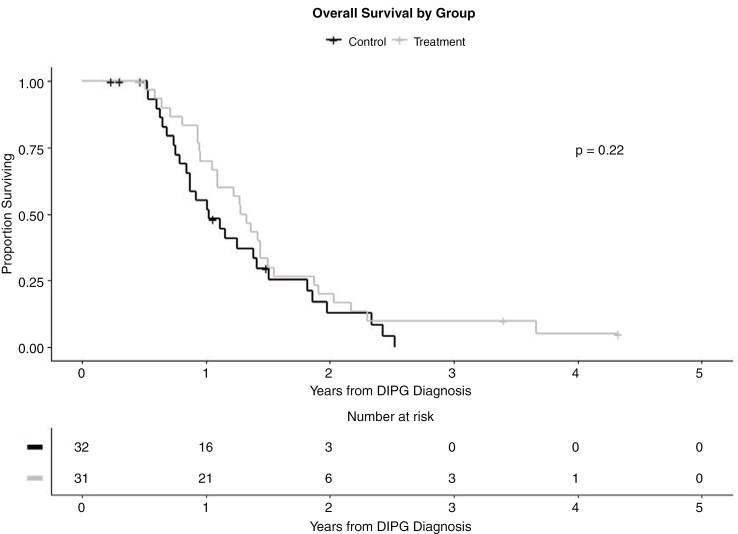
Kaplan–Meier curve of overall survival stratified by CED and control cohort. CED, convection-enhanced delivery.

EPE was the only baseline imaging characteristic that demonstrated a significant difference when comparing the CED-treated and the comparison cohorts (65% vs 88%, respectively; *P* = .03). The OS from date of diagnosis is displayed in Figure 3. OS for the entire cohort was 14.67 months (95% CI 12.16–17.05). No significant difference in OS was found between the CED and comparison groups (15.68 months [95% CI 12.66–17.97] vs 12.33 [95% CI 9.50–16.96], *P* = .22). All patients that died in both cohorts succumbed to neurologic progression/death, with no difference in this clinical endpoint.

### Patterns of Radiographic Relapse

**Figure 4.**
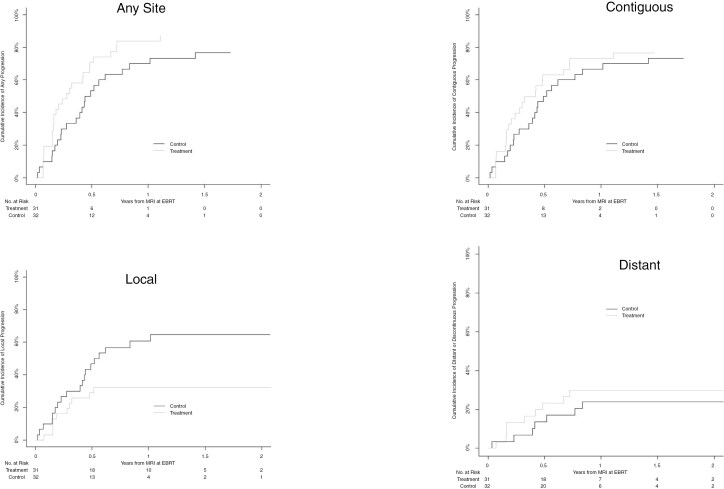
Cumulative incidence curve for progression at any site, local, contiguous, or distant.

**Figure 5.**
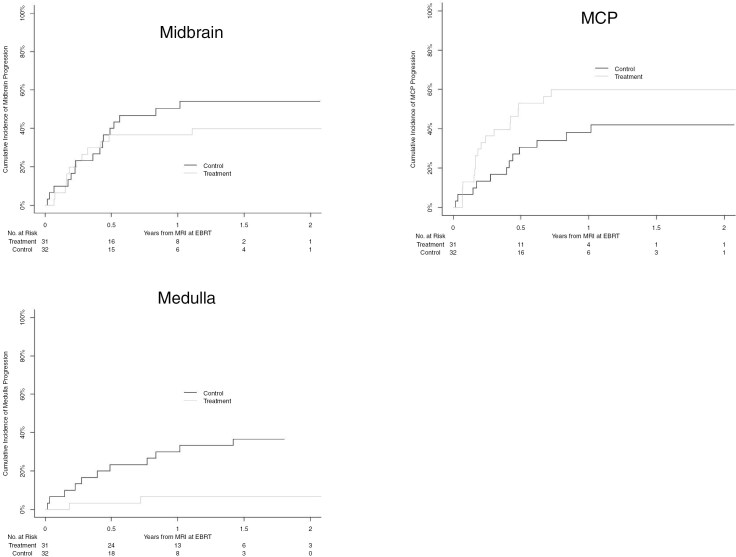
Cumulative incidence curve of contiguous/extrapontine progression subdivided by anatomic sites of midbrain, middle cerebellar peduncles (MCPs), or medulla.

For the entire cohort, the 6-month and 12-month rate of radiographic progression (Figure 4, Table 2) at any site was 62.3% (95% CI 49.9–74.7) and 77.0% (95% CI 66.1–88.0), respectively. Contiguous progression was the most common type of relapse, identified at a 6-month rate of 56.6% (95% CI 43.8–69.3) with a breakdown of midbrain, MCP, and medulla progression (Figure 5) occurring in 38.2% (95% CI 24.3–50.7), 41.9% (95% CI 29.2–54.7), and 13.3 (95% CI 4.6–21.9) at 6 months, respectively. Local progression occurred at a 6-month rate of 37.7% (95% CI 25.4–50.0). Distant progression occurred in 18.4% (95% CI 8.5–28.4) at 6 months and 26.9% (95% CI 15.5–38.4) at 12 months.

**Table 2.** Patterns of relapse progression rates by group

Progression type	Overall cohort 6-month rate (95% CI)	Control group 6-month rate (95% CI)	CED group 6-month rate (95% CI)
Any	62.3 (49.9–74.7)	53.3 (35.0–71.7)	71.0 (54.3–87.7)
Local	37.7 (25.4–50.0)	46.7 (28.3–65.0)	29.0 (12.7–45.3)
Contiguous	56.6 (43.8–69.3)	50.0 (31.6–68.4)	63.1 (45.2–81.0)
Midbrain	38.2 (25.8–50.7)	40.0 (22.0–58.0)	36.5 (18.9–54.1)
Medulla	13.3 (4.6–21.9)	23.3 (7.9–38.8)	3.3 (0.0–9.9)
MCP	41.9 (29.2–54.7)	30.7 (13.6–47.8)	53.1 (34.7–71.5)
Distant/discontinuous	18.4 (8.5–28.4)	13.6 (1.0–26.2)	23.2 (7.8–38.7)

CED, convection-enhanced delivery; MCP, middle cerebellar peduncle.

When examining the relationship of CED treatment on progression (Table 3), patients receiving CED demonstrated higher rates of progression in general, when considering progression at all anatomical sites (HR: 1.79 [95% CI 1.01–3.18], *P* = .047). However, CED treatment was associated with significantly lower cumulative incidence of local/pontine and medullary progression relative to the comparison group (HR: 0.42 [95% CI 0.20–0.92], *P* = .03; HR 0.14 [95% CI 0.03–0.65], *P* = .01, respectively). If adjusting for presence of EPE at diagnosis, the hazard ratios are HR: 0.45 [95% CI 0.21–1.01], *P* = .052 and HR: 0.13 [95% CI 0.03–0.58], *P* = .0008 for local and medulla progression, respectively. No significant differences in progression were identified at other sites (contiguous, midbrain, MCP, or distant/discontinuous) between treatment groups.

**Table 3.** Association between treatment group and progression type

	Any			Local			Contiguous			Midbrain			Medulla			MCP			Distant/discontinuous		
Group	HR	95% CI	*P* value	HR	95% CI	*P* value	HR	95% CI	*P* value	HR	95% CI	*P* value	HR	95% CI	*P* value	HR	95% CI	*P* value	HR	95% CI	*P* value
Control	Ref	Ref	Ref	Ref	Ref	Ref	Ref	Ref	Ref	Ref	Ref	Ref	Ref	Ref	Ref	Ref	Ref	Ref	Ref	Ref	Ref
Treatment	1.79	1.01–3.18	.047	0.42	0.20–0.92	.03	1.32	0.73–2.38	.35	0.73	0.34–1.54	.41	0.14	0.03–0.65	.01	1.83	0.88–3.82	.11	1.40	0.52–3.78	.50
Treatment^a^	1.96	1.07–3.56	.03	0.45	0.21–1.01	.05	1.39	0.75–2.55	.29	0.80	0.37–1.72	.57	0.13	0.03–0.58	.01	1.89	0.88–4.08	.11	1.60	0.58–4.42	.37

EPE, extrapontine extension; MCP, middle cerebellar peduncle.

^a^Adjusted for EPE at diagnosis MRI.

## Discussion

DIPG is a devastating diagnosis with dismal median survival of approximately 8–12 months.^2,3^ Experimental systemic therapies have been studied in numerous phase I/II clinical trials, and while most have demonstrated safety, few have yielded a significant survival benefit with doses limited by systemic toxicity. Combination therapy of bevacizumab, irinotecan, and erlotinib resulted in an OS of 13.8 months and dual inhibition of CDK4/6 and mTOR yielded a median OS of 13.9 months.^7,8^ Surgically targeted delivery of therapy such as with CED can achieve increased local concentration of therapeutics while limiting systemic toxicity. CED in early-phase DIPG trials has demonstrated OS rates of 15.3 months in 2 published small cohorts including the initial NCT01502917 cohort and a trial of the Renishaw Drug Delivery system with repeat infusions of carboplatin/sodium valproate.^15,19^ The median OS observed in our retrospective CED cohort of ~15.7 months, while better than historically defined survival, did not achieve a statistically significant difference between the CED and comparison group. This could be due to several factors including but not limited to the limited effects of a single CED treatment, variability in volume of drug distribution/tumor coverage, or lack of drug efficacy. However, assessing the efficacy of single CED treatment by evaluating OS is likely of only limited value, as a single localized drug delivery cannot necessarily be expected to widely control an infiltrative tumor. As such, a method of evaluating patterns of relapse/failure was needed to determine the efficacy of the local treatment. We proposed an anatomic and radiographic method of evaluating patterns of relapse which was then used to demonstrate a significant difference in rates of initial DIPG progression locally in the pons (6-month relapse CED: 29.0% [95% CI 12.7–45.3] vs comparison: 46.7% [95% CI 28.3–65.0]) as well as in the medulla (6-month relapse CED: 3.3% [95% CI 0.0–9.9) vs comparison: 23.3% [95% CI 7.9–38.8]) following CED treatment. While the conclusions are limited by the retrospective and nonrandomized nature of this study, the defined patterns of relapse can be a vital addition to further CED trials as an outcome measure.

The ultimate goal of any clinical trial in DIPG is to significantly alter the course of disease with an effect of improved clinical status and OS. This study did not demonstrate an improvement in these factors. However, in CED trials, while one can bypass the BBB and shift the drug toxicity profile, efficacy is dependent not just on the drug itself, but also on a variety of factors related to the mechanism of delivery. Different catheter systems, infusion rates, infusion volumes, number of infusions, number of targets, or tumor consistency/presence of necrosis can affect the volume of drug distribution. In a CED trial, one can theoretically deliver an efficacious drug, but be limited by the success/failure of the delivery. If only considering OS at the end of a CED trial, it is possible to misconstrue insufficient drug distribution/coverage as lack of drug efficacy. So, to avoid this misinterpretation, we propose that including anatomic/local progression as an outcome can evaluate whether a drug is demonstrating efficacy within the targeted region. This is perhaps similar in concept to separating the local and distant progression of metastatic lesions treated with stereotactic radiosurgery (SRS). Distant progression outside of the treated SRS field is not in itself a treatment failure, but it requires further intervention to achieve disease control. If a drug delivered by CED demonstrates an effect on local control in the targeted region, then perhaps with further optimization, the next iterations of CED, whether it be through repeat CED procedures,^20^ multiple target sites, larger volumes of distribution,^25^ or alternate delivery systems,^15,26^ can achieve wider drug coverage and therefore disease control and survival. Alternative agents can also be considered.^10,12,15,27^ The antibody included in this trial therapeutically targets and blocks an inhibitory immune checkpoint molecule, and the conjugated diagnostic I-124 radioisotope allowed for tracing drug distribution and clearance. Use of other radioisotopes or drugs delivered by CED may have different outcomes and require similar evaluation. It is important to note that the proposed anatomic/radiographic evaluations on MRI do not necessarily correlate with clinical or performance status. While radiographic measures can be helpful in evaluating disease control, we have not demonstrated that improved radiographic local control in the pons results in an improved clinical symptom/performance outcomes. As future clinical trials are designed, all of these factors should be measured outcomes.

Prior to our described patterns of relapse, recurrence has generally been separated into local or distant/diffuse progression. Studies after radiation and/or adjuvant chemotherapy/bevacizumab have found local recurrence rates ranging between 50%–77%, and diffuse/distant recurrence rates including leptomeningeal disease between 10%–45.5%.^16,28–30^ Tinkle et al. describe the largest cohort of 105 patients after EBRT with a “local” defined anatomic pattern of relapse of 77%; however, this broadly included brainstem, cerebellar peduncles, and cerebellum.^16^ Makepeace et al. utilized a more specific subdivision of anatomic patterns by evaluating location of EPE and demonstrated that these patterns of EPE at diagnosis can correlate with survival.^23^ For our patterns of relapse, we integrated these methods and separated progression into local/pontine, contiguous extrapontine (subdivided into MCP, medulla, and midbrain), as well as distant (leptomeningeal, cerebellum, diencephalon, etc.) sites to allow for specific evaluation of tumor progression/treatment failure. Given the differences in definitions, it is difficult to compare, but the control cohort in this study had local and contiguous progression rates of ~61% and 67%, respectively at 12 months, which does seem consistent with the above, historically described, cohorts. As we hypothesized, a locally delivered CED treatment to the pons demonstrated significant decreased progression only within the pons and perhaps a gravity–diffusion-dependent effect in the medulla. Interestingly, compared to the control group, the patients receiving CED had a higher rate of progression in general, when considering progression at any anatomical site. Since the groups were not matched, it is possible that the patients in the CED trial had more aggressive disease, more frequent imaging in the CED trial may have identified asymptomatic radiographic progression not observed in the control cohort which likely had less standardized image timing, or that the CED treatment could be associated with increased infiltrative disease outside of the treated field.

### Limitations

DIPG remains a rare entity and thus patient accrual at single institutions is difficult and limits study sample size. While our cohort is similar to or larger than prior studies, the small overall number of patients limits our ability to draw definitive conclusions. This pattern of relapse study included CED patients on trial dose level 5 or higher due to the greater likelihood of therapeutic impact at higher dose levels. However, this cutoff is admittedly arbitrary and could introduce selection bias. Furthermore, selection biases in clinical trial inclusion or inclusion/exclusion criteria for the control cohort are limiting in this study design. While the institutional comparison cohort of patients not treated on the NCT01502917 trial allowed for assessment of overall DIPG progression patterns and comparison with our study group, it is limited by the fact that these patients were retrospectively identified, not case–control matched, and may have received a variety of other treatments that cannot be controlled for in this retrospective study. For example, re-irradiation has demonstrated positive impact on interval progression and survival of recurrent DIPG, but this was not controlled for and is beyond the scope of this study.^31–33^ The different rates of EPE at diagnosis for the CED and comparison groups could also be a confounding variable, as these can correlate with differences in survival.^23^ In addition, given that this analysis relies on retrospective review of imaging, the lack of standardized radiographic progression criteria for DIPG, inability to blind radiologists from the treatment group, and nonstandardized timing of interval imaging can be confounding variables in this study.

## Conclusions

DIPG is an aggressive disease with limited treatment options, poor survival, and infiltrative anatomic sites of recurrence and progression. Our data provide a useful evaluation of radiographic, anatomically defined progression rates in DIPG and demonstrate the importance of considering patterns of relapse when evaluating locally delivered therapy. The lower rate of local and medulla progression in the CED cohort demonstrates the potential efficacy of locally delivered therapy; however, the lack of improved OS highlights the need for continued improvement in design of treatment protocols for this highly infiltrative and deadly disease. Future CED or local surgical therapy trials can benefit from including detailed patterns of relapse as a prospective outcome.

## Data Availability

Deidentified data are available upon reasonable request from the corresponding author.
